# Coping Together: A Qualitative Study Exploring the Work of Home Health Care Assistants in Ireland

**DOI:** 10.3390/geriatrics10050128

**Published:** 2025-09-30

**Authors:** Suzanne Cullen-Smith, Aoibheann McKeown, Kevin McKenna, Oonagh M. Giggins

**Affiliations:** 1 NetwellCASALA, Dundalk Institute of Technology, A91 K584 Dundalk, Ireland; suzanne.smith@dkit.ie (S.C.-S.); d00183883@student.dkit.ie (A.M.); 2 Department of Nursing, Midwifery and Early Years, Dundalk Institute of Technology, A91 K584 Dundalk, Ireland; kevin.mckenna@dkit.ie

**Keywords:** home healthcare assistants, caregiver well-being, aging population, community-based care

## Abstract

**Background/Objectives**: Home healthcare assistants (HHCAs) play a vital role in supporting older adults to remain in their homes. Yet, this work is often performed under conditions of emotional strain, limited resources, and systemic undervaluation. This study answers the question, how do HHCAs cope with work-related stress? **Methods:** Undertaken during the COVID-19 pandemic, a period of heightened stress and mandated social distancing, online interviews were conducted with HHCAs (*n* = 10). Data were inductively analyzed and themes were identified. **Results:** It was found that amid experiences of fear, caregiver stress, grief, and exhaustion, HHCAs coped with resource, communication, and care challenges by relying on informal peer-managed communication systems with colleagues. Leveraging existing peer-support coping strategies, HHCAs negotiated caring for others while taking care of themselves alongside a care ecosystem under unprecedented strain. **Conclusions:** HHCAs are increasingly vital to front-line home health care amid global aging and a shift toward community-based services. Urgent organizational reform is needed to support their well-being, prevent stress, and avoid burnout. Research-informed sector-wide planning must ensure adequate resources to maintain high-quality home care in the face of rising demand and anticipated future health crises.

## 1. Introduction

In Ireland, more than 115,000 people aged 65 years and older live alone in private community-based dwellings, a number expected to rise dramatically in the coming years [1]. Home healthcare services play a critical role in helping older adults to remain in their homes for as long as possible, improving physical and emotional health as well as quality of life [2,3,4,5,6]. Introduced in 1972, home care services in Ireland are delivered by home healthcare assistants (HHCAs) who are responsible to conduct a broad range of tasks to support the care of clients, broadly classified as personal, domestic, and social care activities [7]. However, unlike healthcare assistants (HCAs) in institutional settings who have a narrower range of defined responsibilities within a clinical care team, the work of community-based HHCAs is primarily undertaken independently and supervised at a distance by case managers. A surge in demand for home care during the COVID-19 pandemic saw HHCAs taking on additional responsibilities beyond their traditional roles [8]. This shift placed added pressure on HHCAs, who experienced rapid and notable changes in their work. 

The working environment of HHCAs has previously been linked with stress [9]. Lack of autonomy and control over work conditions is a contributor to elevated stress [10,11]. For example, HHCAs’ work environments can be unpredictable, as clients’ homes may vary significantly in cleanliness, safety, and available care resources. Tight schedules set by employers or client needs also result in little flexibility to self-determine how work time is spent [12,13].

Time-pressure in the workplace, considered one of the most challenging aspects of HHCA work, has been linked to both physical strain and emotional stress [14]. Operating with high-level and often vague or unrealistic care plans, HHCAs are expected to independently respond to circumstances as they arise using their initiative and limited training [15]. Yet HHCAs generally lack authorization to act autonomously in the provision of care [6]. Moreover, current education and training has been highlighted as being disconnected from the reality of working as a HHCA, thereby providing inadequate preparation for those who work in home settings [16,17,18,19]. Meanwhile limited interaction with supervisors and little feedback on performance can contribute to a sense of disconnection, low job satisfaction, and workplace stress [12,20].

This research was carried out during the early months of the COVID-19 pandemic, a period that intensified existing stress within home-based care. Findings are presented from a study conducted with HHCAs in Ireland, exploring their experiences of providing care to older adults and the coping strategies they employed to manage stressful work demands and self-care. HHCAs are vital to the care of older adults. High levels of work-related stress have been shown to reduce employee retention rates [21], a concern in the home care sector. Yet, there is a dearth of literature examining how HHCAs cope with work pressures. This paper addresses this deficit by answering the question, how do HHCAs cope with work-related stress?

## 2. Materials and Methods

### 2.1. Study Design

A qualitative research design was employed, using semi-structured one-to-one interviews to gain a deep understanding of the experiences of HHCAs working with older adults.

### 2.2. Participants

A purposive sample of HHCAs, working in paid roles as home/community HCAs, were recruited to take part. Participants were required to be: working as a HHCA with older people in the community for at least six months, fluent in written and verbal English, and aged between 18 and 65 years. A recruitment advertisement was sent to a gatekeeper in home-care organizations (HCOs) (*n* = 75) operating in Ireland, for distribution to HHCA staff. Interested candidates self-identified thus avoiding potential concerns about HCO management influence on the research process. The advertisement was also placed on social media. All candidates (*n* = 10) who responded, met the eligibility criteria. Each received a participant information leaflet (PIL), data protection statement, and a consent form to complete online using Microsoft Forms (Microsoft Corporation, Redmond, WA, USA).

### 2.3. Data Collection

One-to-one semi-structured interviews were conducted online using Zoom (Zoom Video Communications, Inc., San Jose, CA, USA). An interview schedule comprising open-ended questions and prompts guided the discussion around narrowly defined topics. Interviews were audio-recorded, and transcribed verbatim, thus ensuring the participants’ voices were reflected in the data [22]. During data collection, participants were observed for indications of emotional upset and supported to take a break from the interview if distress arose. A list of support resources was provided to all participants in case of post-interview distress.

### 2.4. Data Analysis

Interview transcripts were imported into NVivo 12 software (QSR International Pty Ltd. Burlington, MA, USA, 2018, https://lumivero.com/products/nvivo/ (accessed on 9 December 2024) for inductive thematic analysis, following the six-step framework described by Braun and Clarke [23]. Initially, familiarization with the data was achieved through repeated readings of the transcripts, allowing for an in-depth understanding of the content. A descriptive coding approach [24] was used by A.M.K to generate initial codes from all transcripts and by S.C.S from a selection of transcripts for confirmation. Iterative comparison of defined codes was undertaken (by A.M.K, S.C.S, and O.M.G). Final agreed codes were then organized into potential themes by grouping similar codes together. The themes were subsequently reviewed and refined to ensure they accurately represented the data. Throughout the data analysis process, the authors considered how their positionality might influence the analysis and interpretation of findings (A.M.K, a nurse with 3+ years of work experience as a HHCA; S.C.S, a social science researcher with 5+ years of experience training HHCAs; O.M.G, a physiotherapist and quantitative researcher; K.M.K, an experienced nurse, educator, and healthcare trainer). Theme validation was achieved through discussion and agreement between the researchers.

### 2.5. Ethical Considerations

The protocol for this study was reviewed and approved by the School of Health and Science’s Human Research Ethics Committee in Dundalk Institute of Technology, Ireland (Date of approval 9 July 2020). 

## 3. Results

Interviews were conducted until data saturation was reached. By the eighth interview no new information was forthcoming from participants. Two further interviews were conducted to confirm data saturation. Ten HHCAs (female, *n* = 8; male, *n* = 2) participated in the interviews. Most participants were aged 46 years or older (*n* = 6), Irish (*n* = 7), and had completed second-level education or above (*n* = 9), and half (*n* = 5) had obtained a full Healthcare award at Level 5 on the Quality & Qualifications Ireland (QQI) Framework https://www.qqi.ie/what-we-do/the-qualifications-system (accessed on 9 December 2024).

Two participants were senior carers with the responsibility of care team leaders. Participants were employed in one of three types of HCO: agency-based HCOs (*n* = 6), employed directly by the Irish Health Service (HSE) (*n* = 2), and charity-based HCO (*n* = 2). More than half of the participants (*n* = 6) had worked in some capacity as an HCA for six or more years, with most (*n* = 7) working specifically in a home setting (as a HHCA) for at least one year before the study. For a detailed description of all participants, see Table 1. Pseudonyms are used when quoting participants in the presentation of findings. 

### 3.1. Themes

The thematic analysis of the data identified four overarching themes, namely: the ‘work of a HHCA’, ‘HHCA working experiences’, ‘coping strategies’, and ‘intention to remain working as a HHCA’. This paper focuses on the two themes pertaining to the question of how HHCAs cope with stress: working experiences and the coping strategies employed by HHCAs (Table 2). The remaining themes address a separate research question about HHCA retention, with findings being separately prepared for publication. 

#### 3.1.1. HHCA Working Experiences

HHCAs, as frontline workers, provide care to some of the most at-risk populations. The combination of increased workloads, fear of virus transmission, and emotional fatigue created a uniquely challenging and often overwhelming work environment for those providing home care at the time of data collection. The sub-themes addressed below capture the heightened pressure faced by HHCAs during this time while also reflecting pressures already being experienced prior to the COVID-19 pandemic.

- Stress

The demanding environment of providing home care during the pandemic was highly stressful for participants, who experienced significant increases in physical, emotional, and mental strain. Stress level increases were attributed to additional and rapidly evolving safety protocols, managing client fears, and coping with the uncertainty surrounding the COVID-19 virus: “They [the stress levels] were high before this, but they’re even higher now” (Vicky). Many noted that the challenges of their roles, already demanding before the pandemic, were exacerbated by the new realities of working amidst the global health crisis. Additional interconnected experiences of fear, lost work time, exhaustion, hopelessness, trauma, and their consequences, combined to increase stress to new levels for HHCAs.

- Fear

Fear of the COVID-19 virus was expressed in two primary ways: the HHCAs’ fear of transmitting the virus to others and clients’ fears of contracting it. This dual concern is evident in participant comments: “I don’t want to be going into someone and then I get COVID or pass it on” (Alice). Similarly, participants noted the heightened anxiety of clients, as illustrated by one caregiver who shared: “You’re hyper-conscious if ya [you] cough at all… because the other person [the client] is very nervous, very afraid of picking something up” (Kate).

Pervasive fear led to changes in how caregivers interacted with clients during the pandemic, feeling apprehensive about close physical proximity, an integral part of the caregiving role. As one HHCA recounted: “I used to go in and sit beside a client and talk to them. [Now] I’m constantly running away from them because I’m afraid of if I have it and I give it to them, I couldn’t live with my conscience of killing somebody” (Vicky). Heightened tension, between being responsible to maintain safety and providing care in a personal way, thus added a layer of emotional strain to the existing challenges of providing care.

- Time

The perceived increase in work absences among HHCAs was a further pandemic stressor. Some absences were because of typical illnesses: “I wasn’t very well, so I was off, had to be off for the two weeks because I was unwell” (Alice). However, absences due to widespread closure of schools and childcare services, also represented difficulties only previously encountered during school holidays. One participant described the cascading effects of these challenges: “We had so many people [colleagues] that went out sick or were unable to work because of schools being closed, so they had no childcare in place, [or] because they were unwell themselves and things like that” (Kate). These disruptions not only added to the work of those who remained on duty but also exemplify the precarious balance HHCAs constantly maintain between their professional and personal responsibilities.

- Exhaustion

The extent of stress experienced was evident in descriptions of difficulty unwinding at night, often with intrusive thoughts about work: “Before I even sleep at night, I would be thinking, ‘Who is my client the next day?” (Beth). Poor sleep was a common issue, linked to both mental and physical stress. One HHCA expressed: “I’m not sleeping at night. I go to bed. I can’t knock off. I knock off about half 11 and I’m awake about four, five o’clock with pains all over the bottom half of my body… My body’s tired” (Vicky). Unrelenting stress heightened by the pandemic also impacted HHCAs’ working practices, causing some to struggle daily with the decision to go to work: “I had constant tiredness, no energy, just not wanting to get up some days. There was some days I’d be in bed, and I’d say God, what’ll I ring in and say today? So, some days, I was thinking, oh God, maybe if I said I had quite a high temperature, then I wouldn’t have to go into work and stuff like that” (Anne). 

In addition to the demands of the job, the pandemic left participants feeling emotionally and physically exhausted: “I’m just wrecked. Emotionally, I do cry. I have a great cry now and then. When the burden gets too much” (Paul). These feelings were echoed by another: “I’m physically and mentally drained” (Vicky). Participants also described a cumulative effect, from persistent emotional and physical exhaustion, with fatigue intensifying over time. Although some were able to temporarily manage stress while at work, the full impact was experienced after finishing a shift: “I think it was only afterwards you came to realise how exhausted you were, and you wonder how you did it” (Kate). The physical toll of caregiving under such demanding conditions raised significant concerns about personal health and well-being: “My body’s aching. My body’s sore. I’m existing in this world, but I’m not living. And now with the lockdown, it’s even harder… I’m fed up of my [ill] health. I need to get my health on track. I’m physically and mentally drained” (Vicky).

- Hopelessness

Participants described how work exhaustion impacted their personal lives: “I found, like, in my home life. I was just kind of shutting down. I was exhausted” (Kate). Constant fatigue and stress led to weariness with being ‘on the front line’: “I’m fed up with this pandemic. I’m fed up of driving. I’m fed up of looking at everybody else staying at home. I’m fed up with being a front-liner” (Vicky). The impact of ongoing exhaustion and stress was described by one participant as burnout: “I feel burnt out. I’m just totally burned out. I’m totally wrecked. I’m just drained… just worn out. Just lack of energy, no motivation, just so tired. The least thing, you know, you’d nearly get upset over. [I am] Just so tired.” (Anne).

A consequence for some was feeling hopeless and deflated: “[I feel like] giving up. [I] Just want to stay at home. Why bother? Cry. Just sit and cry. What am I on earth to live for?” (Vicky). Despite these sentiments, all participants continued to attend work and provide client care despite reporting considerable stress levels.

- Trauma

One impact of working during the COVID-19 pandemic was the diminishing effect on emotional and mental welfare: “You’re brought down into, you know, this kind of depression. Everybody’s getting anxiety” (Liz). HHCAs also felt the emotional impact of supporting clients who died: “A client died in my arms. I actually had to do CPR on him. He died in my arms, and the only thing the family wanted to know was, ‘Paul, did he die alone?’ He did not die alone. He died in my arms!’” (Paul). Despite significant experience as an HCA, the effect on Paul was evident during the interview, as he was visibly upset recalling this experience: “I was crying like a baby. Can you see the trauma?” (Paul).

Not having an opportunity to speak about complex cases, with either a member of management or a counselling service, was framed as being inadequately supported by employers: “We’ve watched people die, people, that we’ve cared for. We were never offered counselling” (Vicky). Liz suggested mindfulness training could be valuable, reflecting a lack of mental health support for HHCAs negotiating the trauma and stress impact of working directly with older people during the pandemic: “If they could get something, meditation, anything, something that just keeps their spirits up because once their spirits are up, the people out there that need looking after will be looked after 100%. If you have a carer and they’re feeling down she’s not doing a job to the best ability, she’s not enjoying her job” (Liz). This underscores the reciprocal relationship between HHCA emotional well-being, and the quality of care that can be provided.

#### 3.1.2. Coping Strategies

Participants spoke about coping strategies they employed to manage their own physical and mental well-being, offering insights into how they balanced the demands of their professional responsibilities with the need for self-care.

- Peer Links

Responding to a perceived lack of organizational support during the pandemic, participants employed familiar coping strategies already self-established by HHCAs to mitigate isolated working. Describing working alone as typical, some participants felt isolated: “we’re healthcare workers, and we work very much on our own. You’re very, kind of, isolated in that way. You don’t meet other colleagues, and stuff like that” (Anne). Lone working resulted in a desire for opportunities to increase interaction with peers, including a desire to talk with colleagues about how the work is done, to ensure both quality and safety: “One thing I think is missing. We’re very much on our own. It can be difficult when you’re particularly … [working] with those [client calls] that are very difficult. You have to have somebody for safety, really. You [need to] have some way to check in” (Alice). 

In place of organizationally provided support, HHCAs provided support to each other informally. Despite not being employed as a team leader, Paul described the assistance he offered his co-workers in an almost familial relationship: “I’m treated like I’m the big brother of the carers here because I’m the only man in the group of ladies” (Paul). Taking care of co-workers included sharing information or assisting in any way possible, such as collecting and distributing PPE [Personal Protective Equipment]: “I’m looking out for them. I know the importance of protection [from COVID-19] because of the lungs…” (Paul).

- Group Chats

This sense of mutual support among colleagues was also evident, with informal digital communication channels helping to bridge the gaps in interaction and provide a platform for sharing knowledge and resources. Some clients require the assistance of two HHCAs, referred to as ‘double-up calls’. These calls provided a chance to interact with colleagues. However, not all clients required two carers, and opportunities to meet up with colleagues remained limited. Instead, social media platforms like *WhatsApp*, already in use for some time, allowed HHCAs to stay in contact with peers and continue to share information. Digital group chats were also used to provide peer-supported learning and work task information: “‘Oh, do you not know how to do that? Let me show you’, or if you’re at a client you’ve never been to before, ‘has anyone been here? Does anyone know what I need to do?’” (Becky). 

Participants explained how group chats helped support colleagues in troubleshooting issues that arose during a client visit: “If we don’t know what to do [or] they don’t know how to use a piece of equipment, the group chat comes in handy when we start ringing each other or texting and saying ‘well, this is how I done it or this is how you done it” (Becky). Group chats provided a vital coping tool for HHCAs, both for sharing information on how to do work-tasks and to share work-related updates: “We share all the information. Everything that’s warranted. Proper information about work. Our group is not about talking crap” (Paul). The combination of established peer-support and use of a familiar digital tool proved to be a successful self-directed stress mitigation strategy leveraged by HHCAs during the pandemic. 

## 4. Discussion

The findings outlined above highlight the profound challenges HHCAs face as they care for vulnerable populations in demanding and uncertain environments. Referred to elsewhere as nurse’s aides, healthcare support, or personal support workers, HHCAs continued working despite a society-wide, protective withdrawal to home during the COVID-19 pandemic. During this time, pre-existing pressures were intensified. The pandemic amplified already documented challenges and introduced new stressors. These drained the stress resilience of HHCAs resulting in significant additional emotional, physical, and mental strain. Existing high work-pressure levels were heightened by fears of virus transmission and the burden of increased work demands and trauma. Similar to findings elsewhere [25], the dual fear of contracting the virus and potentially passing it on to clients placed a significant mental toll on HHCAs, contributing to difficulties relaxing outside work hours as well as poor sleep quality. Moreover, HHCAs faced additional financial difficulties during the pandemic, challenges already established as stressful due to the precarious nature of this role [20,26].

This study uncovers the emotional toll that providing care places on HHCAs. Physical and emotional exhaustion, accumulated over time, contributed to hopelessness and affected the ability of HHCAs to maintain motivation and energy in their work. The spillover effects into participants’ personal lives were also notable. A diminished capacity to engage in family and home responsibilities underscored the far-reaching impact of crisis-intensified work-related stress and fatigue. Increased workloads and disrupted workplace dynamics placed a further emotional labour burden on HHCAs. Exacerbated stress has already been established, where HHCAs are required to complete extra tasks as compensation for deficits of social or domestic support previously provided by family members or friends [8]. The emotionally experienced shift from homely to more clinical environments, during the pandemic, was also linked to high levels of stress and burnout compromising physical and mental well-being of care staff [27,28,29].

These experiences during the COVID-19 pandemic help underscore the support needs of essential healthcare workers. A critical buffer to workplace stress is the provision of supportive management processes [9,12]. Already inadequate support, withdrawn further at this challenging time, exacerbated pre-existing inequities long experienced by HHCAs as a marginalized workforce [19]. Indeed, the findings reflect similar sources of stress to earlier studies, such as coping with the trauma of client deaths without adequate organisational supports [20,30]. Yet, this cohort of essential health care workers are already under-represented in policies and initiatives aimed at improving the work experiences of health care personnel. It is critical that the findings presented here are not dismissed as COVID-19 specific or as pandemic-stress induced exaggerations. In this regard it is relevant to highlight that most (*n* = 7) participants had worked for over 12 months as a HHCA, thus representing an informed perspective when comparing early and pre-pandemic work experiences.

**Figure 1:**
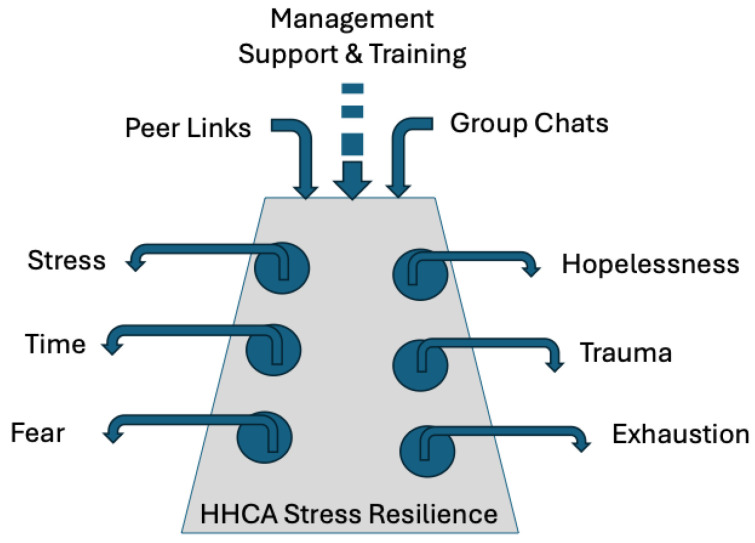
Concept model of factors draining and contributing to stress resilience of HHCAs.

The factors found to contribute to and drain the stress resilience of HHCAs are illustrated in Figure 1. Burnout is a stress syndrome characterized by emotional exhaustion, a diminished sense of personal accomplishment, and hopelessness that negatively affects the stress resilience and retention of health care workers [10,31,32].

The well-being of HHCAs affected by burnout can, in-turn, compromise patient care [33]. Thus, it is essential for organizations and health systems to take proactive steps in stress prevention and the support of HHCAs, both to improve employee health and well-being and to have a positive impact on overall workplace satisfaction. Strategies such as effective supervision and relevant training are necessary for sustained service provision in ‘ordinary’ times. If effectively and routinely implemented, these may provide both a buffer and a structure to support HHCAs in extraordinary times, such as pandemics or other systemic health crises.

HHCAs already used to working alone, before the COVID-19 pandemic, reported increased isolation and loneliness when opportunities to meet with colleagues or members of management were significantly curtailed [34]. However, despite unprecedented pressure and challenges, HHCAs demonstrated remarkable resilience and resourcefulness by adapting several existing coping strategies to the pandemic care provision context. Where sufficient formal organizational support was absent, heavy reliance on peers strengthened the ability to navigate evolving roles and new responsibilities and contain work-related stress. A shared ethos of support enabled HHCAs to both support and depend on colleagues. 

Peer support included teaching and learning from each other in addition to giving and receiving emotional support, as part of a self-established HHCA community. This dependence on peer support is not exclusive to home care settings, but was also evident in long-term care settings [35]. Participants in this study did not represent supporting each other as an additional stressor. Nonetheless, reliance on HHCAs to configure their own (peer) support and training structures is likely to place an undue burden on already pressurized key workers. Systemic failure to address this deficit is short-sighted, limiting the developmental scope and growth potential of a critical sector of the healthcare workforce.

The critical role of collegial peer support in stress and burnout management for HHCAs working alone was highlighted in this study but found to be less preventative than managerial support in pre-pandemic studies [30]. As elsewhere, social media tools and platforms such as WhatsApp were shown to be an invaluable, critical, and accessible communication resource used even more widely by HHCA support networks amid COVID-19 challenging conditions [32]. Our qualitative sample of HHCAs suggests that the perception and experience of workload is closely tied to how the emotional benefits of digital peer support might be harnessed within organizational work processes to manage ongoing job stresses, while aligning with ethical organizational practice requirements. Further exploration is warranted to expand on emerging research demonstrating the mediating relationship between stressful emotional labor and psychological wellbeing of HHCAs [36].

Widespread transferability of the findings is limited due to the sample size and qualitative approach, which emphasised deep understanding of individual experiences over further quantification of broad sectoral trends. Nonetheless, other studies conducted with larger and similar cohorts, and across a range of geographical locations, align with those presented in this paper. HHCAs who did not have access to, or confidence using, social media (to find the recruitment notice) or digital tools (for being interviewed) are not included in this research. Further studies should consider expanding research approaches for wider inclusion.

Finally, the evidence presented suggests that the perception and experience of workload may contribute to manifested stress. Workload was not defined or measured as part of this study. Future research should examine how different stakeholders define workload, both quantitatively and qualitatively, and how differences between expectations and experiences of workload influence stress for HHCAs. Nonetheless, the consistency of findings on stress-related experiences and ongoing support needs, both before and during the COVID-19 pandemic, highlights the need for immediate interventions and policy action to support this critical workforce, as also called for in most previous studies.

## 5. Conclusions

HHCAs are increasingly relied upon for healthcare support at home, against a background of global ageing populations and a push towards community and home care provision. There remains an urgent need for organizational reform to address the well-being of HHCAs and mitigate excessive stress or burnout in this critical cohort of care workers. Furthermore, the findings underscore the need for sectoral policies and planning to ensure necessary HHCA well-being resources are in place to sustain high-quality home-based healthcare services in an era of growing demand and further anticipated pandemics. Further longitudinal research is needed to monitor sectoral and contextual change, track progress of initiatives, and to inform strategic responses to the impacts of expected and unexpected changes to the HHCA work context.

## Figures and Tables

**Table 1:** Participant Demographics.

Pseudonym	Age	Ethnicity	Healthcare Award	HCO Type	HCA (HHCA) Years
Alice	56–60	White	No	Agency	1–5 (1–5)
Anne	46–50	White Irish	Yes	Health Service	10+ (1–5)
Jenna	56–60	Irish	Yes	Agency	6–10 (1–5)
Becky	21–25	Irish	No	Agency	1–5 (1–5)
Frank	60+	Asian	Yes	Agency	1–5 (1–5)
Kate	36–40	White Irish	No	Charity	6–10 (1–5)
Liz	31–35	Irish	Yes	Charity	1–5 (6–10)
Vicky	41–45	Irish	No	Agency	6–10 (6–10)
Paul	51–55	South African	No	Agency	6–10 (1–5)
Beth	56–60	Filipino	Yes	Health Service	6–10 (6–10)

**Table 2:** Two themes and eight sub-themes from HHCA interviews (*n* = 10).

Themes	Sub-Themes
HHCA working experiences	Stress Fear Time Exhaustion Hopelessness Trauma
Coping Strategies	Peer Links Group Chats

## Data Availability

The data presented in this study are available upon request from the corresponding author.

## Abbreviations

The following abbreviations are used in this manuscript:

HCA	Healthcare Assistant
HHCA	Home Healthcare Assistant
HCO	Home Care Organization

## References

[1] Central Statistics Office Older Persons Information Hub, Living Alone. https://www.cso.ie/en/releasesandpublications/hubs/p-opi/olderpersonsinformationhub/housing/livingalone/.

[2] Health Service Executive Home Support Service for Older People. https://www.hse.ie/eng/home-support-services.

[3] Wiles J.L., Leibing A., Guberman N., Reeve J., Allen R.E.S. (2012). The Meaning of ‘Aging in Place’ to Older People. Gerontologist.

[4] Levasseur M., Desrosiers J., Whiteneck G. (2010). Accomplishment level and satisfaction with social participation of older adults: Association with quality of life and best correlates. Qual. Life Res..

[5] Sixsmith A., Sixsmith J. (2008). Ageing in Place in the United Kingdom. Ageing Int..

[6] Kusmaul N., Butler S., Hageman S. (2020). The Role of Empowerment in Home Care Work. J. Gerontol. Soc. Work..

[7] Institute of Public Health in Ireland Improving Home Care Services in Ireland: An Overview of the Findings of the Department of Health’s Public Consultation. https://www.publichealth.ie/reports/draft-regulations-providers-home-support-services-overview-findings-department-healths.

[8] Bell S.A., Krienke L., Brown A., Inloes J., Rettell Z., Wyte-Lake T. (2022). Barriers and facilitators to providing home-based care in a pandemic: Policy and practice implications. BMC Geriatr..

[9] Swedberg L., Chiriac E.H., Tornkvist L., Hylander I. (2013). From risky to safer home care: Health care assistants striving to overcome a lack of training, supervision, and support. Int. J. Qual. Stud. Health Well-Being.

[10] Kim B.J., Ishikawa H., Liu L., Ohwa M., Sawada Y., Lim H.Y., Kim H.Y., Choi Y., Cheung C. (2018). The effects of job autonomy and job satisfaction on burnout among careworkers in long-term care settings: Policy and practice implications for Japan and South Korea. Educ. Gerontol..

[11] Spector P.E. (2002). Employee Control and Occupational Stress. Curr. Dir. Psychol. Sci..

[12] Franzosa E., Tsui E.K., Baron S. (2019). ‘Who’s Caring for Us?’: Understanding and Addressing the Effects of Emotional Labor on Home Health Aides’ Well-being. Gerontologist.

[13] McDonald A., Lolich L., Timonen V., Warters A. (2019). Time is more important than anything else’: Tensions of time in the home care of older adults in Ireland. Int. J. Care Caring.

[14] Andersen G.R., Westgaard R.H. (2013). Understanding significant processes during work environment interventions to alleviate time pressure and associated sick leave of home care workers—A case study. BMC Health Serv. Res..

[15] Franzosa E., Tsui E.K., Baron S. (2018). Home Health Aides’ Perceptions of Quality Care: Goals, Challenges, and Implications for a Rapidly Changing Industry. New Solut. A J. Environ. Occup. Health Policy.

[16] Cavendish C. The Cavendish Review: An Independent Review into Healthcare Assistants and Support Workers in the NHS and Social Care Settings. https://assets.publishing.service.gov.uk/government/uploads/system/uploads/attachment_data/file/236212/Cavendish_Review.pdf.

[17] Conyard K., Codd M., Metcalfe A., Corish S., Flannery J., Hannon P., Rusk B., Yeates S., Bahramian K. Healthcare Assistants and Qualified Carers, A Trained, but Untapped Underutilised Resource: A population-Based Study in Ireland of Skillset, Career Satisfaction, Wellbeing and Change Across All Sectors and Care Settings. https://www.lenus.ie/handle/10147/627406.

[18] Drennan J., Hegarty J., Savage E., Brady N., Prendergast C., Howson V. Provision of the Evidence to Inform the Future Education, Role and Function of Health Care Assistants in Ireland. Report for Health Service Executive. https://www.hse.ie/eng/staff/resources/hrstrategiesreports/health-care-assistant-literature-review-2018.pdf.

[19] Sterling M.R., Cho J., Ringel J.B., Avgar A.C. (2020). Heart Failure Training and Job Satisfaction: A Survey of Home Care Workers Caring for Adults with Heart Failure in New York City. Ethn. Dis..

[20] Smith S., Murphy E., Hannigan C., Dinsmore J., Doyle J. (2019). Supporting older people with multimorbidity: The care burden of home health-care assistants in Ireland. Home Health Care Serv. Q..

[21] Ravalier J., Morton R., Russell L., Rei Fidalgo A. (2019). Zero-hour contracts and stress in UK domiciliary care workers. Health Soc. Care Community.

[22] Creswell J.W., Báez J.C. (2020). 30 Essential Skills for the Qualitative Researcher.

[23] Braun V., Clarke V. (2006). Using thematic analysis in psychology. Qual. Res. Psychol..

[24] Saldaña J. (2021). Coding Techniques for Quantitative and Mixed Data in The Routledge Reviewer’s Guide to Mixed Methods Analysis.

[25] Markkanen P., Brouillette N., Quinn M., Galligan C., Sama S., Lindberg J., Karlsson N. (2021). ‘It changed everything’: The Safe Home Care qualitative study of the COVID-19 pandemic’s impact on home care aides, clients, and managers. BMC Health Serv. Res..

[26] FitzGerald C., Moynan E., Lavelle C., O’Neill C., Robinson K., Boland P., Meskell P., Galvin R. (2024). Exploring the Impact of COVID-19 on Home Care Workers: A Qualitative Study. Gerontol. Geriatr. Med..

[27] Sweeney M.R., Boilson A., White C., Nevin M., Casey B., Boylan P., Staines A. (2022). Experiences of residents, family members and staff in residential care settings for older people during COVID-19: A mixed methods study. J. Nurs. Manag..

[28] Beattie M., Carolan C., Macaden L., Maciver A., Dingwall L., Macgilleeathain R., Schoultz M. (2023). Care home workers experiences of stress and coping during COVID-19 pandemic: A mixed methods study. Nurs. Open.

[29] Howe A.S., Jules K., Tan J.K., Khan R., Li A.K., Edwards B., King E.C., Nizzer S., Gohar B., Yazdani A. (2024). The effects of occupational and mental stress among home care rehabilitation professionals working during the COVID-19 pandemic: An exploratory qualitative study. Home Health Care Manag. Pract..

[30] Boerner K., Gleason H., Jopp D.S. (2017). Burnout After Patient Death: Challenges for Direct Care Workers. J. Pain. Symptom Manag..

[31] Maslach C., Leiter M.P. (2016). Understanding the burnout experience: Recent research and its implications for psychiatry. World Psychiatry.

[32] Willard-Grace R., Knox M., Huang B., Hammer H., Kivlahan C., Grumbach K. (2019). Burnout and Health Care Workforce Turnover. Ann. Fam. Med..

[33] Hall L.H., Johnson J., Watt I., Tsipa A., O’Connor D.B. (2016). Healthcare Staff Wellbeing, Burnout, and Patient Safety: A Systematic Review. PLoS ONE.

[34] Kelleher D., Lord K., Duffy L., Rapaport P., Barber J., Manthorpe J., Leverton M., Dow B., Budgett J., Banks S. (2022). Time to reflect is a rare and valued opportunity; a pilot of the NIDUS-professional dementia training intervention for homecare workers during the Covid-19 pandemic. Health Soc. Care Community.

[35] Titley H.K., Young S., Savage A., Thorne T., Spiers J., Estabrooks C.A. (2023). Cracks in the foundation: The experience of care aides in long-term care homes during the COVID-19 pandemic. J. Am. Geriatr. Soc..

[36] Kuo T.S., Chu L.C., Kao P.L., Shih C.L. (2023). The Effect of Job Satisfaction on Psychological Wel-Being for Taiwanese Home-Care Workers, Mediated by Emotional Labor. Healthcare.

